# Case report: The diagnostic challenge of primary cardiac intimal sarcoma

**DOI:** 10.3389/fcvm.2023.1089636

**Published:** 2023-02-09

**Authors:** Naili Ye, Lan Lan, Huijuan Hu, Jinping Liu, Haibo Xu

**Affiliations:** ^1^Department of Radiology, Zhongnan Hospital of Wuhan University, Wuhan, China; ^2^Department of Cardiovascular Surgery, Zhongnan Hospital of Wuhan University, Wuhan, China; ^3^Hubei Provincial Engineering Research Center of Minimally Invasive Cardiovascular Surgery, Wuhan, China; ^4^Wuhan Clinical Research Center for Minimally Invasive Treatment of Structural Heart Disease, Wuhan, China

**Keywords:** intimal sarcoma, myxoma, cardiovascular magnetic resonance, CMR, multimodality imaging

## Abstract

Primary cardiac intimal sarcoma, an extremely rare cardiac tumor subtype, is often mis-diagnosed owing to its rarity and non-specific clinical and radiological features. We report a case of cardiac intimal sarcoma mimicking atrial myxoma in which the clinical presentation and multimodality imaging are described in detail, and diagnostic challenges are highlighted.

## Introduction

Primary cardiac intimal sarcoma is an extremely rare subtype of cardiac tumor, with only a few such cases having been reported (1–8). It often occurs in the left atrium, whereas it seldom originates in the right atrium and mitral valve (6, 8). Owing to its rarity and non-specific characteristics, cardiac intimal sarcomas are often mistaken for myxomas or thrombi. We report a case of cardiac intimal sarcoma of the left atrium and provide a detailed description of multimodality imaging aimed at highlighting pre-operative diagnostic challenges.

## Case description

**FIGURE 1:**
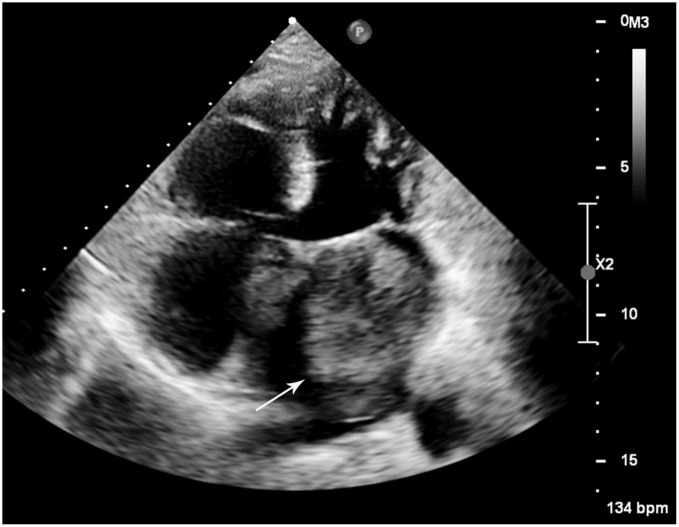
Transthoracic echocardiogram of the left mass. A large hypoechoic mass (white arrow) was revealed in the left atrium attaching to the lateral atrial wall *via* a broad base.

A 52-year-old woman was admitted to our hospital with a 3-month history of progressive cough and shortness of breath. On admission, laboratory findings and non-contrast thoracic computed tomography (CT) scan results were negative for infectious diseases. Her electrocardiogram was normal (heart rate, 76 bpm); however, transthoracic echocardiography revealed an irregularly shaped 4.8 × 6.7 mm hypoechoic mass in the left atrium that appeared broadly based and originating from the lateral atrial wall (Figure 1). The mass induced significant mitral stenosis through prolapsing toward the left ventricle during diastole.

**FIGURE 2:**
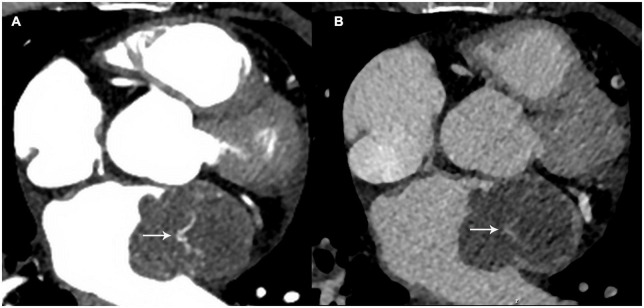
The mass showed heterogeneous enhancement at the arterial phase **(A)** white arrow and the contrast uptake decreased with a longer delay time **(B)** white arrow.

The patient had undergone cervical cancer resection in 2014. No mass or mass-like lesion of the heart had been observed on contrast-enhanced thoracic CT during the follow-up period in 2019. However, possible cardiac metastasis was now suspected. Contrast-enhanced CT and cardiovascular magnetic resonance (CMR) imaging were performed for further characterization. CT images showed that the lobulated mass with homogeneous hypodensity almost occupied the left atrium and the left atrial appendage, extending to the orifice of the left superior pulmonary vein, and showing significantly inhomogeneous enhancement in the arterial phase. Contrast uptake decreased with a longer delay time (almost into the portal venous phase) (Figure 2).

**FIGURE 3:**
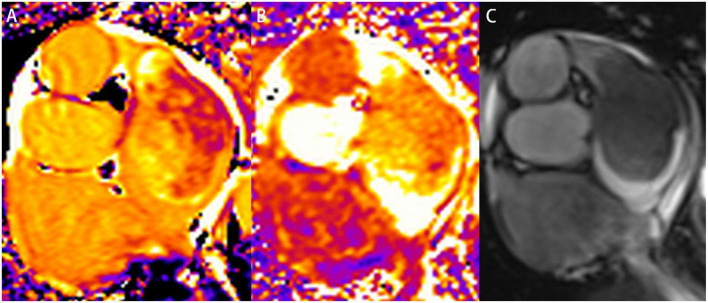
Characterization of the left atrial mass on cardiovascular magnetic resonance imaging. A slice of a short-axis view of left atrium was acquired on native T1 mapping **(A)**, T2 mapping **(B)**, and cine imaging **(C)**, respectively. The mass was heterogeneous on native T1 mapping and T2 mapping. The native T1 value ranged from 1,661 to 2,109 ms. The T2 value was also significantly elevated.

**FIGURE 4:**
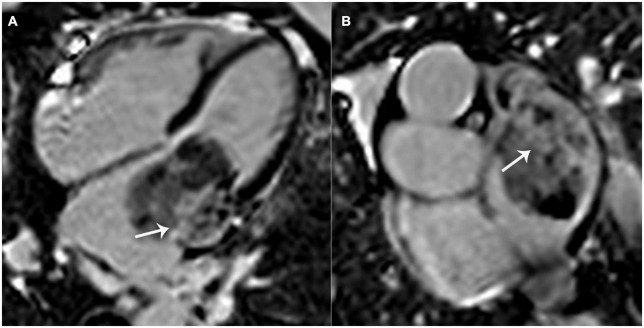
Left atrial mass on late gadolinium-enhanced (LGE) imaging. Significant patchy enhancement (white arrows) was observed on LGE images. **(A)** 4-chamber view; **(B)** a shot-axis view of left atrium.

Regarding CMR imaging, compared with the myocardium, the lesion showed significantly elevated native T1 and T2 values on T1 and T2 mapping (Figure 3). Mildly increased regional perfusion was observed in the mass during first-pass perfusion imaging, as well as heterogeneous enhancement on late gadolinium-enhanced (LGE) imaging (Figure 4). No adjacent tissue infiltration was observed. Radiological findings suggested a possible diagnosis of atrial myxoma.

**FIGURE 5:**
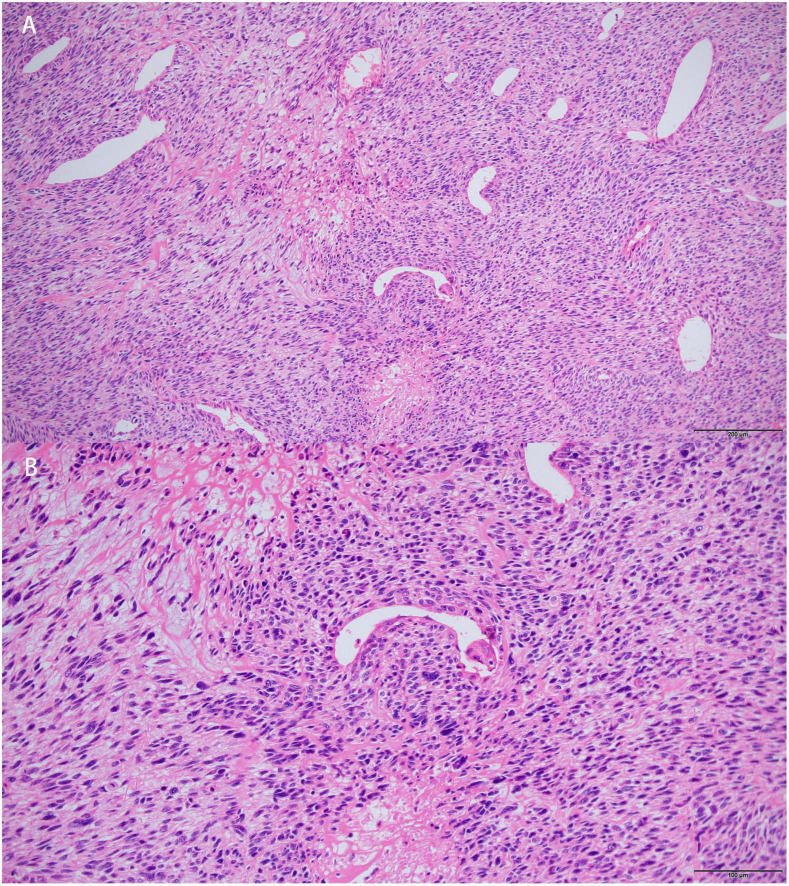
Histopathological features of the left atrial mass. Hematoxylin-eosin (HE) stained section showed that tumors cells were spindle-shaped, prominent atypical. Mitotic activity was frequent. Myxoid changes could be observed in stroma. [Bars: **(A)** 200 μm; **(B)** 100 μm].

Surgical resection was then performed. Histopathological examination revealed spindle-shaped tumor cells with prominent atypia. Mitotic activity was frequent. Some stromal myxoid changes could also be observed in the tumor (Figure 5). Extensive immunohistochemical analyses showed that the cells were positive for vimentin, caldesmon, CDK4, Bcl-2, CD34 (focal), CK (focal), SMA (focal), TLE1 (focal), CD99 (focal) and negative for desmin, EMA, S-100, CD31, ERG, MDM-2, P16, SOX-10, MyoD1, myogenin, STAT6. The Ki-67 proliferative index was 40%. Pathological results supported the diagnosis of high-grade intimal sarcoma. At 4 months post-operatively, the patient was re-hospitalized and underwent systemic chemotherapy for a metastatic lesion of the left femur.

## Discussion

Non-invasive pre-operative assessment of cardiac tumors is vital for further management and could be an important predictor of prognosis. Echocardiography, CT, and magnetic resonance imaging (MRI) are common techniques used for diagnosis and monitoring. Owing to its wide availability and convenience, echocardiography is often used as the first imaging modality to confirm the presence of a cardiac mass and quantify the morphological characteristics, attachment, and mobility of the tumor. CT and MRI are optimal approaches for assessing tissue characterization, vascularity, adjacent infiltration, and extracardiac metastases (9–11). Considering the limitations of different imaging techniques, multimodality imaging of cardiac masses is required for determining treatment strategies.

Intimal sarcoma is a rare subtype of mesenchymal sarcoma that commonly originates in the great vessels with a predilection for the pulmonary arteries (12). The heart is rarely involved in intimal sarcomas, and only a few such case reports have been published (1–4). Cardiac intimal sarcomas are frequently misdiagnosed as either atrial myxomas or thrombi. In this case, the diagnostic challenge has also been highlighted. Typical myxomas are well-defined, often located in the left atrium, and attached to the interatrial septum (9, 10, 13). The mass in our case originated from the atrial free wall, which was the less likely origin of myxomas, as shown in some reported cases (14). In terms of tissue characterization, regarding the similarity of histological components of myxomas and intima sarcomas (4), both may be characterized using isointense/hypointensity on T1-weighted imaging, hyperintensity on T2-weighted imaging and cine imaging, through comparison with the myocardium. Heterogeneous enhancement is often observed in LGE images (9, 10). One point of note is that on CT images, contrast uptake of myxomas is hardly observed in the arterial phase, whereas it significantly increases with a longer delay time (9, 15, 16). The mass in the reported case showed significant heterogeneous enhancement in the arterial phase, and the density decreased with a longer delay time, which is concerning for rich vascularity, an important characteristic of malignant lesions. In addition, a large mass with multiple separated sites of attachment should be considered as a sign of malignancy (1), although this was not observed in our case. Imaging characteristics of the two diseases were summarized in Table 1.

**TABLE 1:** Comparison of imaging characteristics of cardiac intimal sarcomas and myxomas.

	Cardiac intimal sarcoma	Myxoma
Common location	Left atrium	Left atrium > right atrium
	Intracavitary	Intracavitary
Echocardiography	Mobile/Non-mobile mass with single or multiple separated sites of attachment to atrial wall (or atrial septum)	Highly mobile mass being attached to atrial septum (or atrial wall) by a stalk
Contrast-enhanced CT	Hypodensity Heterogeneous enhancement at arterial phase and the contrast uptake decreases with a longer delay time	Hypodensity Calcification may present No obvious arterial-phase contrast enhancement and heterogeneous enhancement is recognized with a longer delay time
CMR		
T1-weighted imaging*	Isointense/Hypointense	Isointense/Hypointense
T2-weighted imaging*	Hyperintense	Hyperintense
LGE imaging	Heterogeneous enhancement	Heterogeneous enhancement

*The signal intensity of T1- and T2- weighted imaging is relative to myocardium. CT, computed tomography; CMR, cardiovascular magnetic resonance.

The mean survival of patients with cardiac intimal sarcoma ranges from 3 months to 1 year (11). Surgical resection is the only management strategy that is associated with prolonged survival (17). Chemotherapy is indicated in patients who cannot undergo surgery, although its benefits are limited.

## Conclusion

Cardiac intimal sarcoma is a rare disease with specific characteristics. This case emphasizes the challenges of differential diagnosis and the importance of multimodality imaging for preoperative assessment.

## Data availability statement

The original contributions presented in this study are included in this article/supplementary material, further inquiries can be directed to the corresponding authors.

## Ethics statement

The studies involving human participants were reviewed and approved by Medical Ethics Committee of Zhongnan Hospital of Wuhan University. Written informed consent for participation was not required for this study in accordance with the national legislation and the institutional requirements.

## Author contributions

LL conducted the draft writing and data analysis. NY was responsible for literature research and data acquisition. HH contributed to image postprocessing. HX and JL supervised the activities. All authors participated in concept development, revision of the manuscript, and read and approved the final manuscript.
